# Albargin Joint Review with Personal Experience

**Published:** 1904-08

**Authors:** 

**Affiliations:** Wurzburg


Translated for Texas Medical Journal.

### Albargin Joint Review With Personal Experience.

BY PROF. DR. SEIFERT, WURZBURG.

Dr. Leibrecht, the discoverer of Albargin, states that it is a combination of silver nitrate with gelatose, a cleavage product of gelatin. It contains 23.6 per cent. of silver nitrate or 15 per cent. of silver, as against 8 per cent. in protargol. It is a faintly yellowish powder, quite coarse and of very light weight, readily soluble in water of any temperature. The reaction of the solutions is quite neutral and dialysis through animal membranes readily occurs. In practice lotions may be prepared for douching, etc., with ordinary drinking water.

Albargin occurs in commerce as a powder or in tablets of 0.2 gm. (3 grs.) each, as that dosage in office hours and in polyclinic work may be facilitated. As the tablets dissolve more slowly than the powder, they must be crushed with a spatula or knife blade upon a sheet of paper before endeavoring to make a solution.

According to researches of Bornemann (*Therapie der Gegenwart*, March, 1901) Albargin destroys gonococci whenever it can reach them, and without the production of undue irritation of the tissues. The average strength of solutions for gonorrhea is 0.2 per cent. If much inflammation is present, the initial strength is 0.1 to 0.15 per cent. At first the albargin solutions are used alone, four or five times daily; later in combination with an astringent, and three times daily. An ordinary gonorrhea syringe is used in the usual manner. If the gonococci disappear permanently from the discharge, the treatment is limited to the use of the astringent. If inflammatory phenomena are not marked at the outset, massive irrigations are begun at once, Diday's method being followed, i. e., the patient receives one such irrigation daily and also gives himself three ordinary injections on the same day.

In cases of first infection, it is more difficult to destroy the gonococci than in second, third, or subsequent infections. In all sixty cases were treated with albargin, twenty-seven first attacks. Of the whole number eighteen developed posterior urethritis with adnexa complications. In the cases of simple anterior urethritis, the gonococcus disappeared in from one to forty days; as a rule, in from three to eight days. A notable advantage of albargin, aside from its dialyzability, is its cheapness.

According to Chrzelitser, who has reported over ninety cases of gonorrhea treated with albargin (*Dermatol. Centralbl., V*), the latter is a very satisfactory antigonorrheal remedy, which, however, like all other drugs, occasionally leaves us in the lurch. The duration of successful treatment may be from six to thirty days. Albargin belongs to those remedies which destroy the gonococcus and at the same time modify the inflammatory changes, which do not irritate, and which by reason of dialyzability are able to exert a deep action.

The experience of Klotz (*Med. News*, November 29, 1902), with albargin in twenty-six unselected cases of gonorrhea, causes him to declare that it comes near being the "ideal antigonorrheic" remedy of Neisser. He believes he may maintain that the urethra becomes rid of gonococci in a relatively short time. In some recent cases the gonococcus actually disappeared after a single injection. He had never observed such results after the use of other silver salts. An injection of 1 or 2 parts per 1000 causes neither pain nor increased secretion.

The observations of Malejew extend over twenty cases (*Military Med. Journal,* St. Petersburg, March, 1902), which had received no treatment before admission to hospital. Injections of 1 to 1.5 per thousand were prescribed to be used twice daily—morning and evening—by the patient himself. The maximum duration of successful treatment was thirty-two days. In a single case the patient recovered in a week. Malejew came to the conclusion that albargin does not irritate the urethral mucosa, that it possesses notable bacteriological powers against gonococci, so that the course of the disease is much abridged; that it is much cheaper than protargol. Complications may appear during the albargin treatment just as they may under any other management of gonorrhea.

Out of forty-three cases of acute gonorrhea, Meyer (*Gaz. in ern. d. med. Prat.,* 1902, 24) cured thirty-four completely and permanently in two or three weeks. In a few cases only it became necessary to complete the cure with sulphocarbolate of zinc. In five cases albargin exerted but little influence over the gonorrheal process, and in four others, relapses occurred, which yielded to albargin and mild astringents. Albargin was used in 0.2 per cent. strength in three daily injections and one irrigation. In thirty-seven cases of chronic gonorrhea, albargin was used in 0.3 to 0.5 per cent. strength. Of this number twenty-five were permanently cured, and five others more or less benefited. In a single case the improvement was slight, and in the remaining five the remedy was of no avail. The length of treatment was from seven to ten weeks. A case of simultaneous gonorrehal cystitis was very effectively managed by 0.1 per cent. albargin. In gonorrheal vaginitis the treatment consisted of irrigation with 0.5 per cent. albargin with simultaneous use of tampons soaked in 0.3 per cent. albargin solution. The results were satisfactory. In four cases of endometritis gonorrhea, two of which were surely acute, one chronic and one doubtful, intrauterine irrigations and tampons saturated with 0.2 per cent. albargin were found to be very useful. Meyer, in view of these results, asserts that albargin is the best known remedy for combating gonorrheal infection. In acute cases, complete recovery was the usual result, while in chronic cases the complete cures amounted to 60 per cent. and cases more or less benefited to 15 per cent.

Pick (*Therapie d. Genenwart,* 1903) treated a large number of cases of gonorrhea with from ¼ to 1 per cent. albargin injections, which were made by the patients themselves three times daily. In-patients also received irrigations of 0.02 to 0.05 per cent. solutions. The gonococcus vanished on the average after eight days treatment, and did not return. According to Pick's experience we possess in albargin an antigonorrhic which is equal to any known in respect to rapidity of result and absence of irritating qualities, and which excels all others in the certainty of its action. We are therefore justified in recommending it in all cases of acute gonorrhea.

Toth (*Orvosi Hetilap.*, 1902, No. 10) treated sixty-five cases of gonorrhea with albargin in the form of Janet irrigations of 0.1 to 0.2 per cent. strength. In the afternoon the patients themselves used injections of 0.2 to 0.5 per cent. strength. With solutions of from 0.1 to 0.5 per cent., there was no irritation of the urethra worth mentioning; the discharge soon diminished and the gonococci were killed. Good results are to be expected in acute and subacute cases; while in chronic urethritis the desired end is attained more rapidly by the use of more stimulating remedies.

To abort gonorrhea, Blaschko (*Berliner Klin. Woch.*, 19, 1902), uses upon the first day 1 to 2 per cent. solutions of albargin, while upon the following days one-half per cent. strength is used, the solution being allowed to remain in the urethra three minutes. Frank (*Berliner Klin. Woch.* 19, 1902), uses irrigations of albargin in 0.1 strength to abort gonorrhea, using them on three consecutive days, one irrigation daily, one-half liter each. He emphasizes that albargin costs 10 per cent. less than protargol, and is not decomposed in hot solutions.

I have myself used albargin for six months as an antigonorrhic, and can confirm throughout the statements of the authors cited. Some fifty cases, acute and chronic, were under treatment, all dispensary patients. In the acute cases, I used daily urethral irrigations of 0.1 per cent. strength, while in the chronic type the irrigations were of double strength, but used only twice a week. In none of my cases was it my good fortune to see the gonococci vanish during the first day; in fact, my best result was the disappearance of the germs by the eighth day. Extension of the disease to the posterior urethra, and the development of complications like prostatitis and epididymytitis occurred with the same apparent frequency as under other methods of treatment. Of the chronic cases, about 10 per cent. proved refractory to albargin. I must concede, however, that albargin is an extremely valuable addition to our materia medica for the treatment of gonorrhea, that strong solutions cause relatively little irritation, that it possesses a manifest deep action, and is very much cheaper than protargol.

